# Associations between intraoperative factors and surgeons’ self-assessed operative satisfaction

**DOI:** 10.1007/s00464-019-06731-z

**Published:** 2019-03-18

**Authors:** Sofia Erestam, David Bock, Annette Erichsen Andersson, Anders Bjartell, Stefan Carlsson, Karin Stinesen Kollberg, Daniel Sjoberg, Gunnar Steineck, Johan Stranne, Thordis Thorsteinsdottir, Stavros Tyritzis, Anna Wallerstedt Lantz, Peter Wiklund, Eva Angenete, Eva Haglind

**Affiliations:** 1 Department of Surgery, Institute of Clinical Sciences, Sahlgrenska Academy at University of Gothenburg, Sahlgrenska University Hospital, SSORG - Scandinavian Surgical Outcomes Research Group, Östra Campus, Gothenburg, Sweden; 2 Institute of Health and Care science, University of Gothenburg, Gothenburg, Sweden; 3 Department of Urology, Skåne University Hospital, Malmö, Sweden; 4 Section of Urology, Department of Molecular Medicine and Surgery, Karolinska Institutet, Stockholm, Sweden; 5 Division of Clinical Cancer Epidemiology, Department of Oncology, Institute of Clinical Sciences, Sahlgrenska Academy, Gothenburg, Sweden; 6 Department of Epidemiology & Biostatistics, Memorial Sloan Kettering Cancer Center, New York, NY USA; 7 Department of Urology, Institute of Clinical Sciences, Sahlgrenska Academy at Gothenburg University, Gothenburg, Sweden; 8 Faculty of Nursing, University of Iceland and Landspitali – The National University Hospital of Iceland, Reykjavík, Iceland; 9 Center for Minimally Invasive Urological Surgery, Athens Medical Center, Athens, Greece; 10 Department of Urology, Icahn School of Medicine at Mount Sinai, New York, NY USA

**Keywords:** Intraoperative factors, Surgical satisfaction, Self-assessment, Surgical performance, Surgeon, Prostate cancer

## Abstract

**Background:**

Little is known concerning what may influence surgeon satisfaction with a surgical procedure and its associations with intraoperative factors. The objective was to explore the relationships between surgeons’ self-assessed satisfaction with performed radical prostatectomies and intraoperative factors such as technical difficulties and intraoperative complications as reported by the surgeon subsequent to the operation.

**Methods:**

We utilized prospectively collected data from the controlled LAPPRO trial where 4003 patients with prostate cancer underwent open (ORP) or robot-assisted laparoscopic (RALP) radical prostatectomy. Patients were included from fourteen centers in Sweden during 2008–2011. Surgeon satisfaction was assessed by questionnaires at the end of each operation. Intraoperative factors included time for the surgical procedure as well as difficulties and complications in various steps of the operation. To model surgeon satisfaction, a mixed effect logistic regression was used. Results were presented as odds ratios (OR) with 95% confidence intervals (CI).

**Results:**

The surgeons were satisfied in 2905 (81%) and dissatisfied in 702 (19%) of the surgical procedures. Surgeon satisfaction was not statistically associated with type of surgical technique (ORP vs. RALP) (OR 1.36, CI 0.76; 2.43). Intraoperative factors such as technical difficulties or complications, for example, suturing of the anastomosis was negatively associated with surgeon satisfaction (OR 0.24, CI 0.19; 0.30).

**Conclusions:**

Our data indicate that technical difficulties and/or intraoperative complications were associated with a surgeon’s level of satisfaction with an operation.

Diverging reports exist regarding the surgeons’ self-assessed satisfaction with a surgical procedure and possible relation to intraoperative factors or outcome measures. Few studies have been conducted on the subject but a recent review concluded that surgeons could be capable of evaluating their own technical skills accurately even though variations between self- and external assessments have been reported [1]. On the contrary, others have reported that surgeons had a limited ability to accurately assess their surgical performance, compared with external assessment [2, 3]. However, the assessment of satisfaction does not reflect performance per se and may as such be a valid measure. In a study on hernia repair, surgeons reported a higher level of satisfaction with open compared with laparoscopic hernia repair and in the same study surgeons reported a relationship between the level of intraoperative frustration and patient outcomes, with higher levels of frustration noted during laparoscopic procedure than during open hernia repair [4].

Surgeon satisfaction could be related to good teamwork as a satisfied surgeon should be less likely than a dissatisfied surgeon to show disruptive behavior in the operating room [5, 6]. The effect of surgeons’ disruptive behavior in the operation room has been described as a shift in attention from the patient to the surgeon and increased mistakes during the surgical procedure [7, 8].

For many years, retropubic open radical prostatectomy (ORP) was the surgical approach for treating localized prostate cancer [9]. About 15 years, ago robot-assisted laparoscopic prostatectomy (RALP) was introduced to improve surgical outcomes and results from the prospective LAParoscopic Prostatectomy Robot Open (LAPPRO) trial revealed a small benefit over ORP regarding erectile dysfunction [9].

We hypothesize that the surgeons’ self-perceived satisfaction of the surgical procedure is associated with intraoperative difficulties and complications.

The aim was to evaluate if surgeons’ self-assessed satisfaction with his/her performance of a prostatectomy (ORP and RALP) was associated with intraoperative difficulties or complications.

## Materials and methods

The LAPPRO trial is an open, non-randomized, prospective, controlled study of open (ORP) and robot-assisted laparoscopic (RALP) radical prostatectomy in which patients from fourteen centers in Sweden were included during 2008–2011 [9, 10]. Primary endpoint for the LAPPRO trial was urinary incontinence 1 year after surgery and was reported in 2015 [9]. Secondary endpoints were erectile dysfunction, oncological outcome, quality of life, and cost-effectiveness [9–11]. After inclusion, patients were followed during the initial 24 months by the use of seven clinical record forms (CRF) and four questionnaires, as described in detail earlier [10]. The intraoperative CRF were answered by respective surgeon, collected by the research nurse at each hospital, and sent to the trial secretariat.

### Ethics

The LAPPRO trial was approved by the Regional Ethical Review Board in Sweden (EPN Dnr 277-07). The trial was registered at ISRCTN (ISRCTN06393679).

### Objectives

The objectives were to explore relationships between intraoperative factors such as difficulties and complications and surgeon satisfaction with the operation.

### Intraoperative variables

Information on the possible predictors, the intraoperative factors such as surgical difficulties or intraoperative complications related to specific steps of the operation, time in the operating room, intraoperative bleeding, performed nerve preservation, that is of the neurovascular bundles, and lymph node dissection were collected in the perioperative CRF. Planned nerve preservation was collected in the preoperative CRF. ORP does in general require less operative time than RALP. Likewise, the amount of bleeding is in general higher during the former procedure. Therefore, in the analyses of operative time and bleeding, different cut-off points were used for ORP and RALP. Prolonged operative time was defined as > 90 and > 180 min for ORP and RALP, respectively. Extensive bleeding was defined as > 500 and > 100 ml, respectively. The perioperative CRF was filled out by the operating surgeon directly after each surgical procedure, see Table 1.

**Table 1:** Association between intraoperative factors and surgeon satisfaction

Report from surgery	Surgeon’s satisfaction*				
	Satisfied (*N* = 2905)	Dissatisfied (*N* = 702)	Comparison	Chance of being satisfied OR(95% CI)	*P* value
Operative method					
ORP	705 (81)	167 (19)	ORP vs. RALP	1.36 (0.76; 2.43)	0.2961
RALP	2200 (80)	535 (20)			
Difficulties with dissection of the urinary bladder?					
Some or severe difficulties	356 (66)	181 (34)	Some or severe difficulties vs. No difficulties	0.62 (0.48; 0.78)	< 0.0001
No difficulties	1865 (84)	359 (16)			
Not applicable	10 (77)	3 (23)			
Not stated	674	159			
Lymph node dissection performed					
Yes	495 (79)	131 (21)	Yes vs. No	0.89 (0.69; 1.15)	0.3717
No	2404 (81)	571 (19)			
Not stated	6				
Difficulties with dissection between bladder neck and the prostate?					
Some or severe difficulties	1040 (68)	486 (32)	Some or severe difficulties vs. No difficulties	0.26 (0.21; 0.33)	< 0.0001
No difficulties	1852 (90)	214 (10)	Some or severe difficulties vs. Not applicable	0.46 (0.08; 2.63)	0.3831
Not applicable	10 (83)	2 (17)	No difficulties vs. Not applicable	1.75 (0.31; 10)	0.5274
Not stated	3				
Difficulties with dissection of seminal vesicles, left side?					
Some or severe difficulties	949 (66)	488 (34)	Some or severe difficulties vs. No difficulties	0.24 (0.2; 0.3)	< 0.0001
No difficulties	1952 (90)	212 (10)			
Not stated	4	2			
Difficulties with dissection of seminal vesicles, right side?					
Some or severe difficulties	942 (66)	490 (34)	Some or severe difficulties vs. No difficulties	0.25 (0.2; 0.31)	< 0.0001
No difficulties	1914 (90)	205 (10)			
Not stated	49	7			
If there was a lobus tertius, was the dissection difficult?					
Some or severe difficulties	316 (69)	139 (31)	Some or severe difficulties vs. No difficulties	0.52 (0.35; 0.78)	0.0014
No difficulties	330 (83)	68 (17)	Some or severe difficulties vs. Not applicable	0.52 (0.4; 0.68)	< 0.0001
Not applicable	2246 (82)	494 (18)	No difficulties vs. Not applicable	1.01 (0.71; 1.42)	0.9752
Not stated	13	1			
Were there any difficulties regarding dissection of the left neurovascular bundle from the prostate?					
Some or severe difficulties	805 (68)	383 (32)	Some or severe difficulties vs. No difficulties	0.14 (0.1; 0.2)	< 0.0001
No difficulties	1165 (94)	72 (6)			
Not applicable	311 (78)	86 (22)			
Not stated	624	161			
Were there any difficulties regarding dissection of the right neurovascular bundle from the prostate?					
Some or severe difficulties	769 (69)	341 (31)	Some or severe difficulties vs. No difficulties	0.2 (0.15; 0.27)	< 0.0001
No difficulties	1161 (93)	83 (7)	Some or severe difficulties vs. Not applicable	0.88 (0.67; 1.17)	0.3850
Not applicable	345 (75)	115 (25)	No difficulties vs. Not applicable	4.36 (3.06; 6.24)	< 0.0001
Not stated	630	163			
Were there any difficulties when the anastomosis was sewn?					
Some or severe difficulties	704 (64)	401 (36)	Some or severe difficulties vs. No difficulties	0.24 (0.19; 0.3)	< 0.0001
No difficulties	2193 (88)	289 (12)			
Not stated	8	12			
Complication with the urethra					
Yes	5 (50)	5 (50)	Yes vs. No	0.14(0.03; 0.62)	0.0092
No	2853 (81)	653 (19)			
Not stated	47	44			
Complication with the anastomosis					
Yes	141 (63)	83 (37)	Yes vs. No	0.17 (0.12; 0.25)	< 0.0001
No	2723 (82)	586 (18)			
Not stated	41	33			
Complication with the intestines					
Yes	70 (73)	26 (27)	Yes vs. No	0.57 (0.34; 0.96)	0.0356
No	2785 (81)	638 (19)			
Not stated	50	38			
Complication with technical equipment					
Yes	90 (70)	42 (30)	Yes vs. No	0.3 (0.2; 0.47)	< 0.0001
No	2757 (82)	624 (18)			
Not stated	50	36			
Complications due to variations in anatomy					
Yes	255 (68)	119 (32)	Yes vs. No	0.27 (0.2; 0.37)	< 0.0001
No	2616 (82)	563 (18)			
Not stated	34	20			
Other complications					
Yes	130 (68)	61 (32)	Yes vs. No	0.32 (0.22; 0.46)	< 0.0001
No	2740 (82)	615 (18)			
Not stated	35	26			
Operating time (min) RALP					
Median (Q1; Q3)	179 (150; 215)	193 (158; 246)	180-min vs. 0–180 min	0.32 (0.24; 0.45)	< 0.0001
0–180 min	955 (85)	164 (14.66)			
180-min	908 (80)	233 (20)			
Not stated	337	138			
Bleeding (ml) RALP					
Median (Q1; Q3)	100 (50; 200)	200 (100; 300)	100-ml vs. 0–100 ml	0.27 (0.21; 0.34)	< 0.0001
0–100 ml	1257 (90)	143 (10.21)			
100-ml	860 (74)	298 (25.73)			
Not stated	83	94			
Operating time (min) ORP					
Median (Q1; Q3)	86 (73; 126)	114 (84; 151)	90-min vs. 0–90 min	0.45 (0.24; 0.86)	0.0152
0–90 min	341 (89)	44 (11)			
90-min	275 (78)	76 (22)			
Not stated	89	47			
Bleeding (ml) ORP					
Median (Q1; Q3)	500 (350; 745)	750 (400; 1200)	500-ml vs. 0–500 ml	0.26 (0.16; 0.41)	< 0.0001
0–500 ml	363 (89)	47 (11)			
500-ml	326 (74)	117 (26)			
Not stated	16	3			
Nerve preservation planned before surgery					
Yes ambition	481 (22)	1736 (78)	Yes ambition vs. No ambition	0.80 (0.60; 1.08)	0.1445
No ambition	100 (20)	394 (80)			
Can’t answer	34 (10)	305 (90)	Yes ambition vs. Can´t answer	0.63 (0.40; 1.01)	0.0570
Not stated	87	470			
Nerve preservation performed					
Yes	2281 (81)	544 (19)	Yes vs. No	1.09 (0.85; 1.39)	0.4823
No	622 (80)	158 (20)			
Not stated	2				
Fulfillment of planned nerve preservation					
Not as planned	87 (65)	47 (35)	Not as planned vs. As planned	0.39 (0.25; 0.60)	< 0.0001
As planned	2133 (80)	517 (20)			
Not stated	685	138			
Number of intraoperative difficulties/ complications					
0	820 (28)	32 (5)	One additional complication**	0.60 (0.56; 0.63)	< 0.0001
1	459 (16)	39 (6)			
2	465 (16)	64 (9)			
3	378 (13)	75 (11)			
≥ 4	783 (27)	492 (70)			

*Row percent within brackets

**The odds of being satisfied after reporting one further intraoperative difficulty or complication

### Surgeon satisfaction

The study outcome, the surgeon’s perceived satisfaction with the surgical procedure, was gained from a question in the perioperative clinical record form (CRF): “How satisfied are you with the performed surgical procedure technically?” with response options in four categories “Not satisfied,” “A little satisfied,” “Quite satisfied,” and “Very satisfied.” The responses were dichotomized into two groups “Not satisfied” (category 1 and 2) and “Satisfied” (category 3 and 4).

### Statistical analysis

**Fig. 1:**
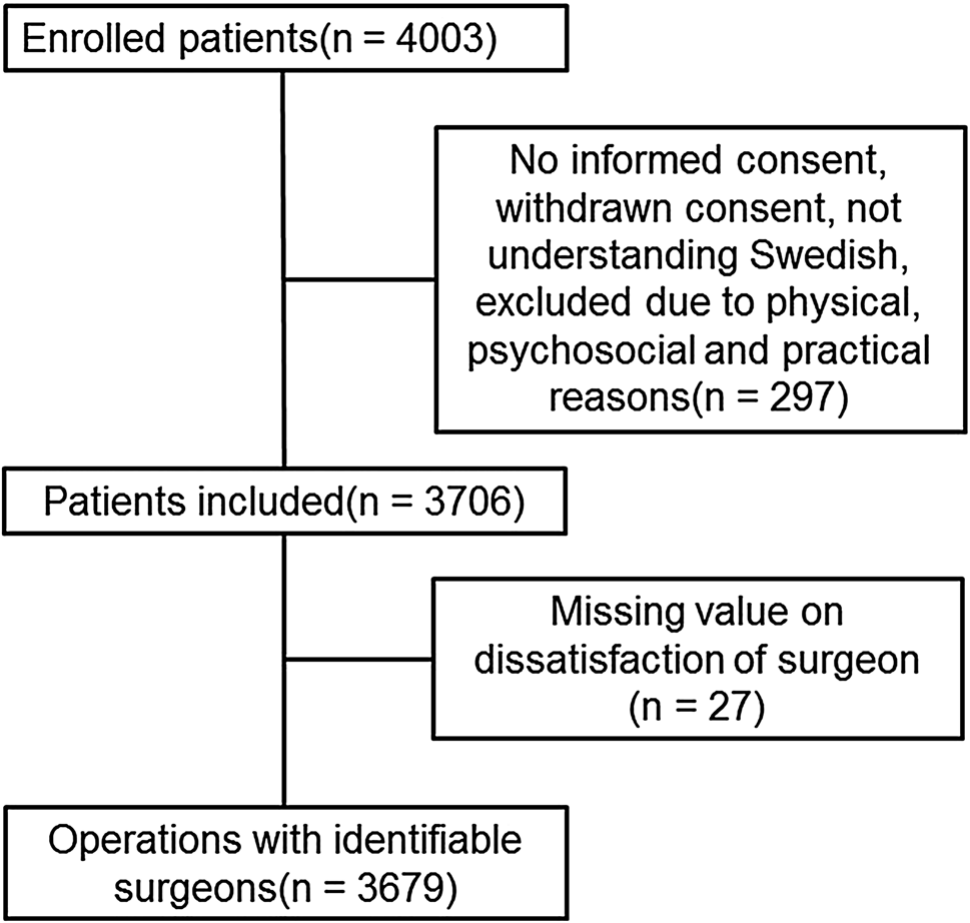
Patients included in the LAPPRO trial

The sample size of the LAPPRO trial was calculated to compare ORP and RALP regarding incontinence at 12-month follow-up [10]. In the analysis of the relationship between intraoperative difficulties and complications and surgeon satisfaction, a mixed effect logistic regression was used where the intra-surgeon dependency was accounted for by a random intercept and with a variance component covariance structure. In order to adjust for differences between surgical procedures as well as for disease severity, type of surgical procedure (ORP and RALP), tumor stage (pathologist T stage), and prostate weight (gram) were included as covariates. In the analysis of operating time and amount of bleeding, ORP and RALP was analyzed separately. The model was fit separately for each intraoperative factor. In addition, the relationship between the number of intraoperative difficulties and complications and surgeon satisfaction was assessed using the same statistical model. Results are presented as odds ratios for satisfaction with 95% confidence intervals. The cohort consisted of all patients included in the LAPPRO trial with intraoperative data available (Fig. 1).

No imputation of missing values was made. The GLIMMIX procedure in SAS® was used for the statistical analyses.

## Results

The LAPPRO trial recruited 4003 patients. Patients included in our analysis are further described in Fig. 1. ORP was performed in 922 patients and RALP in 2757 patients. Sixty-nine identifiable unique surgeons performed 3679 surgical procedures included in the final analyses (Fig. 1). The number of operations performed by non-identifiable surgeons was 27. The number of surgical procedures performed per surgeon within the LAPPRO trial ranged from 1 to 281. For all surgeons, the majority of surgeries were performed with the same technique. The surgeons were satisfied with the procedure in 2905 (81%) of the cases and dissatisfied at 702 (19%) occasions (Table 1).

### Intraoperative factors

We found no relationship between surgeon satisfaction and type of surgical technique (OR 1.36, 95% CI 0.76; 2.43), see Table 1.

We found strong associations between surgeon satisfaction and reported intraoperative technical difficulties and complications where satisfaction decreased with the number of intraoperative unwanted events. A surgeon without any reported intraoperative difficulties and/or intraoperative complications was dissatisfied in five percent of the procedures. In 298 operations, the surgeon reported four or more difficulties or complications; only 10 percent of those surgeons were satisfied with their procedure. Each additional reported intraoperative difficulty or complication decreased the odds of being satisfied (0.60, 0.56; 0.63). Surgeons that experienced complications due to variations in anatomy were dissatisfied in 32% of the operations (0.27, 9.2; 0.37). Difficulties with suturing of the anastomosis rendered a dissatisfied surgeon in 36% of the cases as compared with 12% in the absence of suturing difficulties (0.25, 0.20; 0.3). Difficulties with dissection of the urinary bladder resulted in a dissatisfied surgeon in 34% of the surgical procedures while only 16% of the surgeons were dissatisfied when there was no difficulties reported (0.62, 0.48; 0.78). Some 30% of the surgeons were dissatisfied with the surgical procedure when complications with the technical equipment were reported (0.3, 0.20; 0.47). This pattern was found for all different types of surgical difficulties or intraoperative complications (Table 1).

If the preoperative plan was to preserve the neurovascular bundles, the surgeon fulfilled this plan in 95% of the cases. Neither planned nor performed neurovascular preservation was associated with surgeon satisfaction. Among surgeons who preoperatively planned to preserve the neurovascular bundles and preoperatively fulfilled their ambition, 80% was satisfied. Surgeons who did not fulfill their preoperative plan on nerve preservation were satisfied in 65% of the cases (0.39, 0.25; 0.60). (Table 1) Among surgeons performing RALP, satisfaction was associated with a median surgical procedure time of 179 min, whereas the median time was 193 min for cases with a dissatisfied surgeon rendering an increased risk for dissatisfaction after a prolonged surgical procedure time (> 180 min; 0.32 (0.24; 0.45)). For surgeons performing ORP, the median surgical procedure time was 86 min for satisfied surgeons vs. 114 min for dissatisfied surgeons (> 90 min; 0.45 (0.24; 0.9)) (Table 1).

Median blood loss during RALP operations was 100 ml and 200 ml among satisfied and dissatisfied surgeons, respectively, rendering an increased risk for dissatisfaction after increased bleeding (> 100 ml: 0.27 (0.21; 0.34)). In the ORP group, blood loss was 750 ml for dissatisfied and 500 ml among satisfied surgeons, respectively (Table 1).

## Discussion

In this study, we found a relationship between intraoperative factors such as surgical difficulties and complications and surgeon satisfaction after radical prostatectomy. Surgeon’s satisfaction was associated with various types of self-reported difficulties and intraoperative complications, as well as with the frequency.

Measurement of surgical performance is important when it comes to improving the quality in surgical treatment [12]. Surgical quality can be measured in many ways and one common measure has been mortality, either as postoperative mortality or in oncologic surgery as long-term survival [12]. However, postoperative mortality may be inappropriate as probably many other factors than “surgical quality” contribute to postoperative mortality and difficult to use as marker of quality due to the low incidence even after extensive surgery [12].

Earlier when surgeon’s frustration and satisfaction during and after surgery was studied, frustration was found to be a better predictor of surgical outcomes. Frustration was related to difficult anatomy in 42% of the cases and thereafter to personnel, equipment, instruments, or technical issues [4]. One might be able to find a connection between surgeon frustration and intraoperative complications and difficulties. We found that 32% of the surgeons were dissatisfied after reporting complications due to variations in anatomy.

Preservation of the neurovascular bundles is desirable as it has been shown to reduce erectile dysfunction [13] and urinary incontinence [14]. We analyzed whether fulfillment of preoperatively planned nerve preservation was associated with increased surgeon satisfaction. We hypothesized that if the surgeon planned to preserve the neurovascular bundles and was unable to do so it would possibly affect the satisfaction report. However, surgeons who had the preoperative plan to preserve the neurovascular bundles and fulfilled their plan were significantly more satisfied than surgeons with the same preoperative plan who had to change their ambitions intraoperatively and could not preserve the nerves.

Assessment of technical skills may be difficult regardless of whether it is made by self -assessment or by peers or faculty [2]. However, it should be possible to use surgeon satisfaction as a starting point for discussions about technical difficulties during a surgical procedure, aiming for gradual improvement. Our results may have clinical implication in the sense that surgeon satisfaction could have an impact on surgical performance by affecting teamwork and operating time, directly, indirectly, or as a combination. We found an association between prolonged operative time and dissatisfaction, which might be an indication that there could be of interest to test models of competent support, as one possible way to improve outcomes and decrease operative time. The association between prolonged operative time and surgeon dissatisfaction is presumed to go both ways as surgeon dissatisfaction should affect the perioperative environment and therefore intraoperative communication and teamwork as a satisfied surgeon should be less likely to show disruptive behavior in the operating room [6, 7, 15]. Our findings of a pronounced relationship between surgeon satisfaction and the number of reported unwanted intraoperative difficulties or complications encourage a discussion on how to handle surgeon dissatisfaction during the surgery. Our results revealed that surgeon satisfaction decreased with the number of reported unwanted intraoperative difficulties or complications. In 298 operations, the surgeons reported four or more difficulties or complications, only 10 percent of those surgeons were satisfied with their procedure. This information in combination with the result that every reported intraoperative difficulty or complication decreased the odds of being satisfied could initiate a discussion on how to handle surgeon dissatisfaction during surgery. What is the reaction of a surgeon who meets setback after setback? Do operative setbacks affect a surgeon’s self-confidence? And if so, could there be ways to handle this situation where a dissatisfied surgeon possibly underperforms leading to further mistakes.

The influence of teamwork in the operating room was not included in this study, although both technical skills as well as non-technical skills could possibly affect surgeon satisfaction. Non-technical skills in the medical setting could include situation awareness, decision making, teamwork, leadership, coping with stress, and managing fatigue [16]. Interventions to improve teamwork have been studied, such as the use of checklists [16, 17]. Related to those are safety climate and risk-management, where focus on the individual has changed to focusing on the system [18].

The surgeon was satisfied with the procedure performed in a majority of cases. The fact that more than twice as many dissatisfied surgeons reported problems due to technique or surgical equipment indicates that their dissatisfaction may in part have been related to teamwork factors as one of the tasks for non-surgeons on the team is to make sure that the equipment is in working order.

The aim of this study was to analyze intraoperative surgical difficulties and complications and their association with surgeon satisfaction. A possible association between surgeon satisfaction and long-term outcomes and functional disorders will be analyzed separately.

One strength of this study is the prospective design and the large study cohort. Further, the procedure was standardized including the detailed intraoperative CRF filled out by the surgeon, detailing a large number of steps of the procedure. A further strength was that surgeons were pseudonymous when answering the perioperative CRF. Only the local investigators could decode the personal surgeon code used, and the local investigators did not have access to results. Limitations in this study were that the question “How satisfied are you with the performed surgical procedure technically?” only covered a certain aspect of the relationship between satisfaction with a surgical procedure and intraoperative factors. Structured observations, video recordings, complementing questions and interviews, and intraoperative record forms addressed to other members of the surgical team might have given more depth in the analyses of surgeon satisfaction. The current statistical model has limitations. It does not account for the full hierarchical structure of the study design as a random center effect could not be included in the model due to computational difficulties. Furthermore, the order in which each surgeon performed the operations was not accounted for. Since there are no repeated measures on the patients, their effects cannot be separated from measurement error, potentially rendering a situation similar to temporal pseudoreplication [19].

## Conclusions

Our data indicate that technical difficulties and/or intraoperative complications were associated with a surgeon’s level of satisfaction with an operation.
